# Effect of self-etching primer/adhesive and conventional bonding on the shear bond strength in metallic and ceramic brackets

**DOI:** 10.4317/medoral.17024

**Published:** 2011-07-15

**Authors:** Behnam Mirzakouchaki, Soodabeh Kimyai, Mahboubeh Hydari, Shirin Shahrbaf, Parvin Mirzakouchaki-Boroujeni

**Affiliations:** Orthodontic Department, TMSU Tabriz, Iran

## Abstract

Introduction: Bracket debonding from the tooth surface is a common problem in fixed orthodontics. The aims of the present study were to assess the bond strength and failure sites in two ways of bonding technique, with metallic and ceramic brackets. Material and Methods: One hundred premolars were assigned to 4 groups of 25 each: Group A, metallic brackets/ conventional procedure; Group B, metallic brackets/Transbond XT; Group C, ceramic brackets/conventional procedure; and Group D, ceramic brackets/Transbond XT. Transbond XT composite paste was used for bracket bonding and cured by conventional light-cure device. Specimens were subjected to thermocycling. One week after bonding shearing force was applied to the bracket-tooth interface. Bonding failure site optically examined using a stereomicroscope under 10 × magnifications and scoring was done using the adhesive remnant index (ARI). Data were subjected to analysis of One-way variance, Tukey post hoc, Chi-square and Spearman’s tests. Results: Mean bond strength (in MPa) were: group A=9.2, group B=8.5, group C=6.2 and group D=5.7. Bond strength differences between groups A and B, and between C and D were not significant, (p<0.0005). Insignificant difference found in ARI in all groups. Conclusion: The bond strengths of metallic brackets were significantly higher than ceramic ones and the selfetching primer produce fewer bonds than the conventional method (clinically acceptable). A positive correlation found between changes in shearing bond strength and ARI.

** Key words:** Acid etching, adhesive remnant index, orthodontic brackets, self-etching primer, shearing bond strength.

## Introduction

 Conventional adhesive system use 3 different agents an enamel conditioner, a primer solution, and an adhesive resin and to bond orthodontic brackets to enamel (1,2). A unique characteristics of some new bonding systems in operative dentistry is that they combine the conditioning and priming agents into a single acidic primer solution. Combining conditioning and priming into a single step improves bonding time and reduce the number of steps during the bonding procedure and result in cost-effectiveness to the clinician and indirectly to the patient with similar bond strengths or even higher bond strengths. 

Transbond Plus is a self-etching primer system (SEP), in which the primer and the conditioner have been mixed together; in this system the risk of contamination with saliva has decreased to a minimum since there is no need for irrigation during the procedure, and the system has a low technique sensitivity. Furthermore, this new technique has some other advantages compared to the conventional system, including ease of the bonding and debonding procedures and a decrease in the time required for adhesive removal (1). 

There is considerable controversy over the bond strength of brackets in the self-etching primer system compared to the conventional bonding system and have reported that bond strength in the self-etching primer system is very low but still much higher than the conventional system. Paskowsky (3) did not demonstrate any statistically significant differences between the bond strengths in the Transbond Plus system and the conventional system. Bishara et al. (4) has claimed that bond strength in the self-etching primer system is higher compared to the conventional system. The differences in the results of studies may be attributed to differences in selecting specimens, type of brackets, bracket retention mechanism, debonding procedures and the type of the adhesive used. 

The use of self-etching primer system for orthodontic purposes has not completely been evaluated. Metallic brackets, made of stainless steel, are the most commonly used brackets but the metallic appearance of brackets is not acceptable for (most) patients. As a result of patients’ desire to use tooth-colored brackets (5), the purpose of the present study was to investigate the relationship between the shear bond strengths of orthodontic metallic and ceramic brackets to enamel, with a conventional etch/priming techniques or a SEP system, subsequent to thermocycling and one week incubation period before testing. The hypothesis to be tested is whether there is a difference in the mean shear bond strength between the use of a conventional multistep or SEP and when they were used either orthodontic metallic bracket or ceramic ones.

## Material and Methods

 The teeth

One hundred freshly human maxillary premolars extracted within a six-month period, for orthodontic reasons in an age range of 18-25 were collected and examined macroscopically for following inclusion criteria: included intact buccal enamel, no carious lesions, no attritions, no cracks caused by extraction forceps, no restorations, no congenital anomalies, no structural defects and no pretreatment chemical agents (e.g., hydrogen peroxide) used on them (2). The teeth were immersed in distilled water at room temperature until used in the experiment. Twenty-four hours before use, all of them stored in 0.2% (weight/volume) Thymol for infection control. At no stage in the investigation were the teeth allowed to dehydrate. The teeth were carefully cleaned with a hand scaler and water-pumice slurry in dental prophylactic cup for 10 seconds. The teeth were of equal occlusogingival and mesiodistal dimensions which were measured using an orthometer with 1 mm variation, (7-8 mm). The teeth were randomly assigned to 4 groups of 25 teeth each and treated as follows:

Group A, metallic brackets and the conventional technique; 

Group B, metallic brackets and the self-etching primer technique;

Group C, ceramic brackets and the conventional technique; 

Group D, ceramic brackets and the self-etching primer technique.

 The Brackets

Fifty orthodontic maxillary metal brackets (Roth 022, Ovation, GAC International, Inc.) and fifty orthodontic maxillary ceramic brackets (Roth 022, Allure III, GAC International, Inc.) were used in this study. The brackets had mechanical retention and the average metal bracket base surface area was determined to be 12 mm2, and the average ceramic brackets base surface area was 12.2 mm2. 

 The Bonding Procedure

The teeth were dried with a gentle current of oil- and moisture-free air for 3 seconds (2,4). On the buccal surfaces of 25 teeth a surface area corresponding to the base surface area of the brackets was marked with a mesh to prepare identical surface areas for acid etching. Then Transbond Plus (3M / Unitek, Morovia, Calif.) containing both the acid and the primer was placed on the enamel for 15 seconds and gently evaporated with air (2,5).

The same marking procedure was carried out on another set of 25 teeth and the buccal surfaces were etched for 15 seconds using 37% phosphoric acid gel (3M, Dental Products, St. Paul Mn 55144) according to the manufacturer’s instructions; followed by rinsing the surfaces for 15 seconds using a water spray. A mild blast of air on the surface for l0 seconds removed any excess water; result in white chalky appearance on enamel surfaces. Then, Transbond XT (3M / Unitek, MIP) liquid primer was applied to the enamel surfaces with a brush and left for 20 seconds; a strong blast of air on the surface for 5 seconds removed any excess primer (2).

Transbond XT (3M / Unitek) adhesive paste was placed on the bracket base and on the prepared tooth surfaces similar to clinical situations. Each bracket on tooth surfaces was subjected to a 300-gr compressive force with a force gauge (Correx, Bern, Switzerland) for l0 seconds to achieve a uniform thickness of the adhesive on all the tooth surfaces (2), and then excess bonding resin was removed with an explorer (4). 

The specimens were light-cured using a conventional halogen source (Astralis 7, Vivadent, Ivoclar). The tip of the light-curing source had a diameter of 8 mm and a constant a light intensity of 400 mw/cm2. The source tip was placed 2 mm away from the surface, delivering the curing light for 40 s in the ceramic brackets (l0 s each from the mesial, distal, occlusal and gingival directions) and for 20 s (10 s each form the mesial and distal directions) in the metallic brackets for sufficient polymerization. The specimens were placed in an incubator at 377 C then to simulate oral conditions they were submitted to a thermocycling regimen of 1000 cycles between 5±2 - 55± 22 C water baths. Dwell time was 30 seconds, with a 10 seconds transfer time between baths, and water bath temperature was be held constant. 

 The Debonding Procedure

For the debonding procedure, the specimens were embedded in acryl in plastic rings. A mounting jig was used to align the facial surface of each tooth to be perpendicular with the bottom of the mold. Each tooth was oriented with the testing device as a guide, so that its labial surface was parallel to the force during the shear strength test, permitting the bracket base to be parallel to the direction of the force. This allowed a shear force to be applied to the bond interface (between the bracket and the tooth). 

A stainless steel rod with a flattened end of 0.5 mm2 cutting edge diameter was attached to the crosshead of a testing machine (Zwick/ Roell, Model 2020). One week after bonding an occlusogingival load was applied at the bracket–tooth interface, producing a shear force at the enamel–adhesive interface in a manner that the resin cut surface was perpendicular to the horizon (2), until the bracket sheared from the tooth. Shear bond strengths were measured at a crosshead speed of 0.5 mm/min. A computer, electronically connected to the testing machine, recorded the force to debond the bracket in Newtons. The bond strength was calculated in megapascals (MPa) by dividing the force in Newtons to the surface area of brackets in (mm2), yielding the result at MPa. 

 Remnant Adhesive

The bond failure sites were examined optically using a stereomicroscope under (Olympus, SZx 9) 10x magnification (2,6), and scoring was done using the adhesive remnant index (ARI). The Adhesive Remnant Index (ARI) consists of a 5-point scale from 1 to 5 as follows:

Grade 5 indicates that no composite has remained on enamel surface.

Grade 4 indicates that less than 10% of the composite is remaining on enamel surface.

Grade 3 indicates that more than 10% but less than 90% of the composite has remained on enamel surface.

Grade 2 indicates that more than 90% of the composite is remaining on enamel surface.

Grade 1 indicates that all the composite has remained on enamel surface and no composite is visible on the bracket.

 Statistical Analysis of Data

One-way ANOVA was applied to compare the shearing bond strengths between the groups. A pairwise comparison within groups was analyzed with the Post Hoc Tukey test. Chi-square test was used to compare ARI between the groups and Spearman‘s correlation test examined the relationship between the shearing bond strengths and ARI. Statistical significance differences were considered at p< 0.5.

## Results

 Shearing Bond Strengths of the Groups

The mean shearing bond strengths in groups A, B, C and D were 9.20 ± 1.41 (6.70 - 11.70) MPa, 8.50 ± 1.10 (6.50 - 10.50) MPa, 6.20 ± 0.16 (4.60 - 7.80) MPa and 5.70 ± 0.68 (4.40 - 7) MPa, respectively.

Comparison of Shearing Bond Strengths in the Groups Tested

One-way ANOVA test demonstrated a statistically significant difference in the mean shearing bond strengths between the four experimental groups [p < 0.0005, f (3, 96) = 66.65].

**Figure 1:**
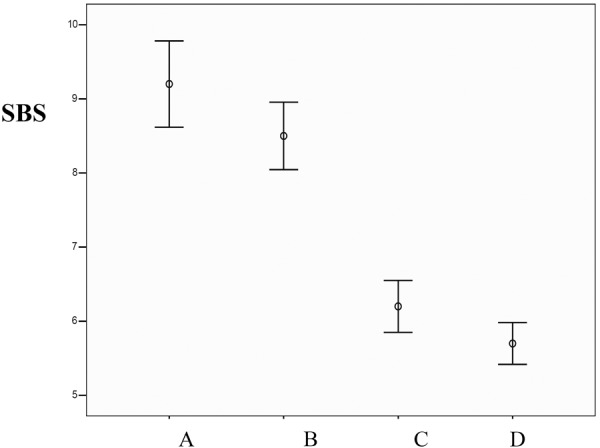
Comparison of shearing bond strengths between the groups.

A Post Hoc Tukey test did not demonstrate any statistically significant difference in the mean shearing bond strengths between groups A and B (p = 0.091). In addition, the test did not indicate any differences between groups C and D in this respect (p = 0.336). ( Table 1and Fig. 1).

Comparison of Remnant Adhesive Index (RAI) between the Groups 

**Figure 2:**
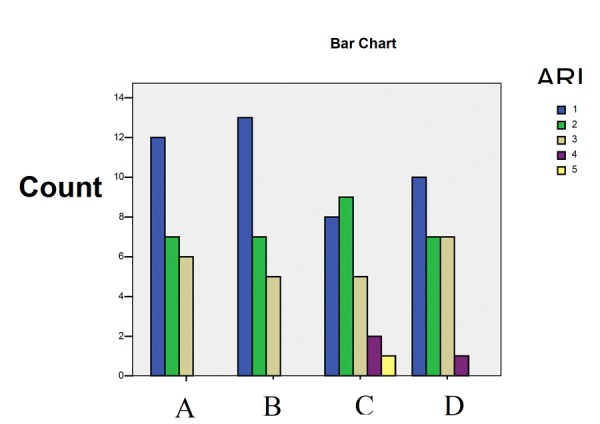
Comparison of ARI between the groups.

Chi-square test did not demonstrate any statistically significant differences in ARI between the groups (p = 0.71, df = 12, x2 = 8.91). ( Table 2 and Fig. 2).

The Relationship between Changes in Shearing Bond Strengths and ARI in the Experimental Groups

Spearman‘s rho demonstrated a statistical relationship between changes in shearing bond strengths and ARI in the experimental groups. This correlation in groups A, B, C and D was 0.82, 0.53, 0.62 and 0.78, respectively. This indicates that changes in bond strengths are parallel with changes in ARI ( Table 3). 

 Debonding 

In the entire experimental groups base on chi-square test, debonding had mainly taken place in the bracket–adhesive interface or inside the adhesive itself. During debonding a part of the adhesive had remained on the bracket and a part had remained on the tooth surface ( Table 4). No cases of bracket failure were observed.

**Table 1:**
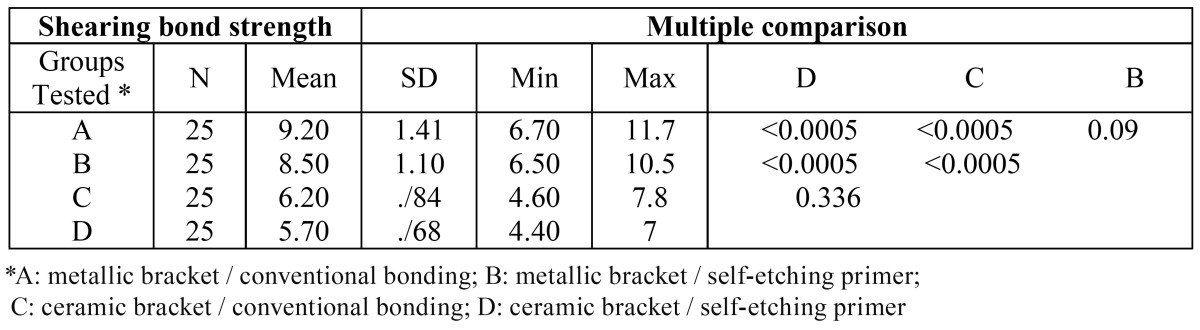
Comparison of shearing bond strengths in the experimental groups.

**Table 2:**
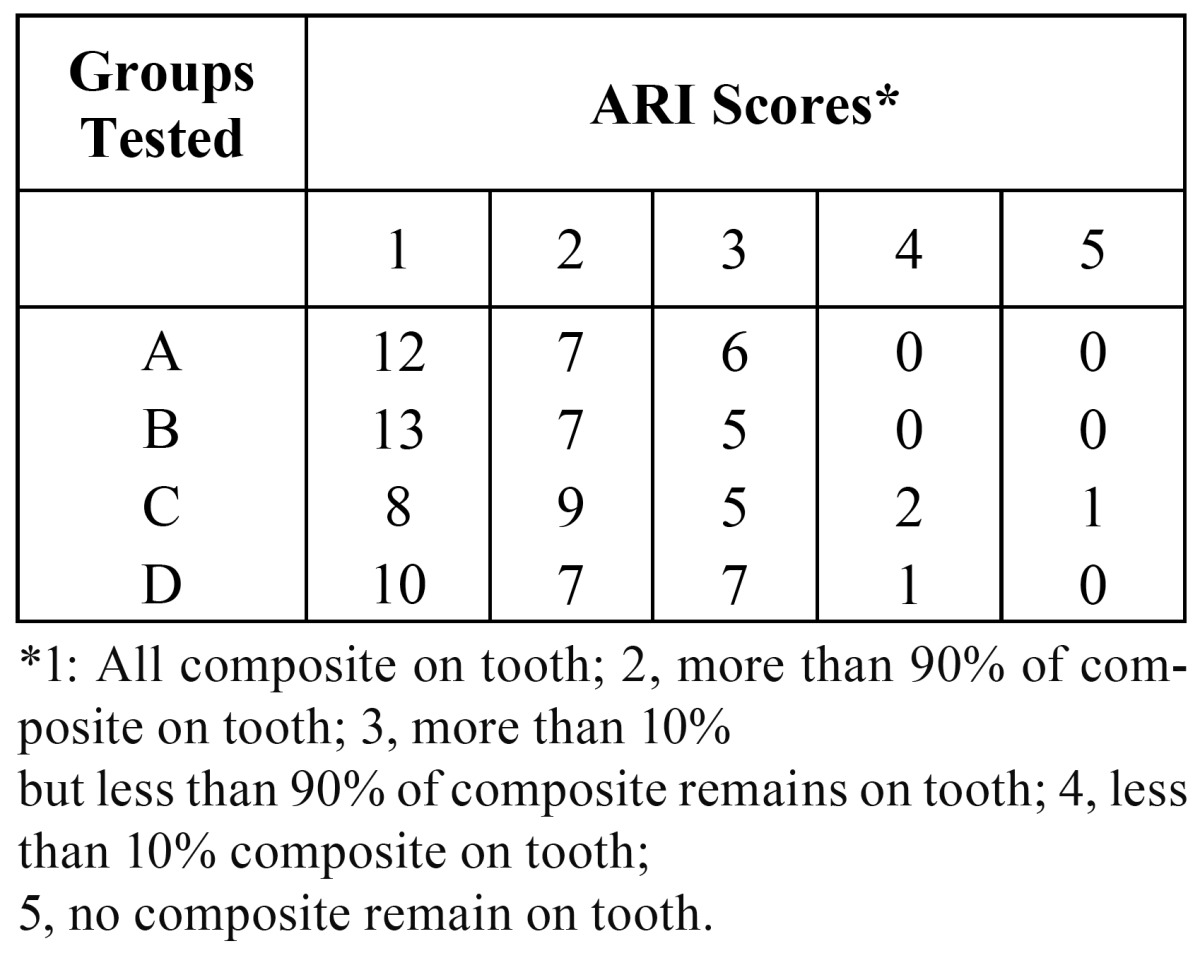
Comparison of ARI between the groups.

**Table 3:**
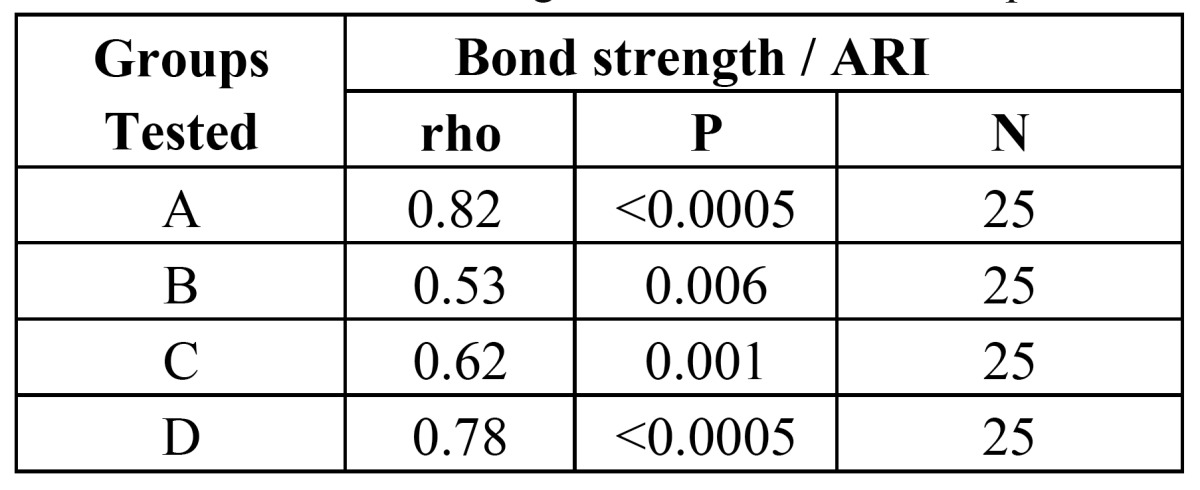
Shear bond strength and ARI relationship.

**Table 4:**
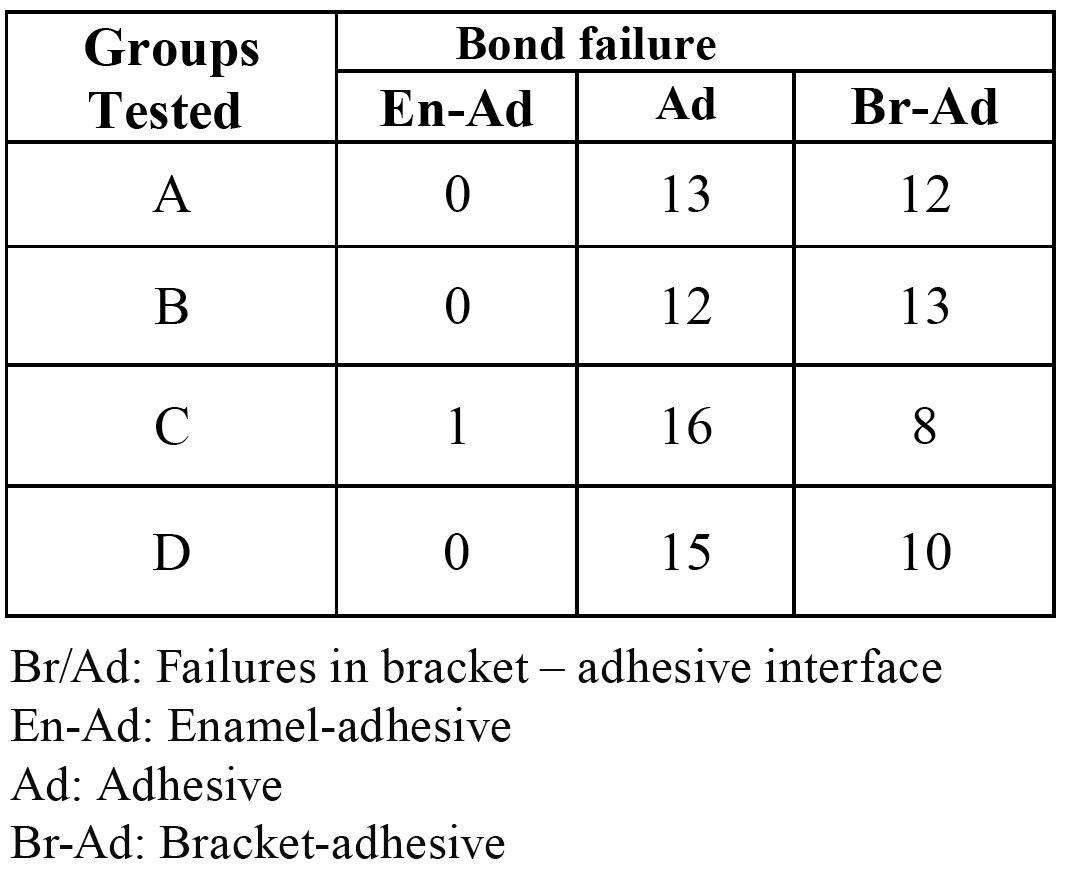
The comparison of the type of debonding in the experimental groups.

## Discussion

The direct bonding of orthodontic brackets has revolutionized and advanced the clinical practice of orthodontics. However, there is a need to improve the bonding procedure by saving time and cost. Although recent bonding systems have been proven reliable, improvements are still necessary to minimize technique sensitivity and reduce the chair time by decreasing the number of steps during the bonding procedure. Traditionally, using acid etchants followed by a primer was an essential part of the bonding procedure of composite adhesives, to allow good wetting and penetration into the enamel surface. 

The use of the new self-etch primers for orthodontic purposes has not yet been fully evaluated. In general, they are thought to simplify the clinical handling of the adhesive systems by combining the etchant and the primer in 1 application. The present study, which was carried out to evaluate the shearing bond strengths of metallic and ceramic bracket with conventional bonding technique versus self-etching primer technique, demonstrated mean shearing bond strengths of 9.20 , 8.50 , 6.20 and 5.70 MPa in groups A, B, C and D , respectively. The values in all the cases were higher than the minimum values and lower than maximum values recommended by various authors (7). 

 Bond Type

According to the results of the present study , there was no statistically significant difference between the mean shearing bond strengths of metallic and ceramic brackets bonded using the conventional technique and self-etching primer procedure ( p= 0.091 in metallic brackets and p=0.336 in ceramic brackets). The results of this study are con-sistent with the results of studies carried out by (1,3,8-11).According to these studies the shearing bond strength of the conventional method is higher than that in self-etching primer method, with no statistically significant differences between the two methods. The results of those studies are different from the results of studies carried out by (2, 12-16). According to these studies the bond strength of the conventional method is significantly greater than the self-etching primer method. (4,6) reported that the bond strength of the conventional method is less than the self-etching primer method.

The difference in bond strengths described by various researchers might be attributed to differences in the selection of specimens (human or animal teeth and anterior or posterior teeth), study design (in vitro versus in vivo), surface preparation, the use of different kinds of adhesives, debonding techniques, the time lapse between bonding and debonding and finally the storage conditions of the teeth during the study period. In a study carried out by Bishara et al. (2) the bond strength of self -etching primer technique (7.1 MPa) was significantly less than that in the conventional technique (10.4 MPa) but it was clinically acceptable. The difference between the results of the present study and that study might be attributed to the lack of thermocycling procedure in that study and differences in the brackets used. In that study only metallic brackets were used and thermocycling procedure was not included in the study design. A great advantage of the present study was the inclusion of a thermocycling procedure in the study design to simulate oral conditions and also the comparison of metallic and ceramic brackets after a week.

 Bracket Type

The shearing bond strength of metallic brackets is significantly greater than ceramic brackets with mechanical retention (8.5 MPa in metallic brackets and 5.7 MPa in ceramic brackets using Transbond Plus and 9.2 MPa in metallic brackets and 6.2 in ceramic brackets using the conventional bonding technique), coinciding with the results of a study, 13.2 MPa in metallic brackets and 8.8 MPa in ceramic brackets with mechanical retention (9). In the present study despite the use of different bonding agents, bond strengths were similar in two bracket types. 

The results of this study with respect to the comparison of metallic brackets and ceramic brackets with mechanical retention do not in accordance with the results of studies carried out by other authors (4,9,17).In the studies carried out by Korbmacher et al. (9) and Kuang Liu et al. (17) no statistically significant differences between the bond strengths of these brackets were found (8.8 MPa in ceramic brackets and 8.7 MPa in metallic brackets). In the study carried out by Bishara et al. (4) the bond strengths of metallic brackets (Victory Series) were less than the ceramic brackets with mechanical retention (Clarity). The differences in the results might be attributed to differences in the adhesives and bonding agents used, the method or the appliance of force application, the duration of force application, the type of the brackets and the inclusion of thermocycling procedure or its absence in the study design.

 Adhesive Remnant Index (ARI)

ARI is clinically important because as the potential of debonding towards enamel and the adhesive increases and less composite is left on tooth surfaces, thus more stress will be applied to enamel surface.

The results of the present study are coincident with the results of studies carried out by other authors (2,9,6,18). 

(6) reported that remaining adhesive on tooth surface in the conventional technique is greater than that in the self-etching primer technique using commercial products of Clearfil SE Bond and Etch & Prime 3.0, whereas remaining adhesive using Transbond Plus was similar to the conventional technique (6).Therefore, bonding type influences the results. In the present study, similar to the conventional technique, Transbond Plus had a lower ARI, which is different from the results of a study carried out by Hosein et al. (19). They reported a higher ARI in the conventional technique compared to self-etching primer technique with metallic brackets (20).

The differences in the results might be attributed to differences in bracket type, variations in bracket base design, the type of the bonding material, the type of the adhesive used and the method or the appliance of force application.

 Bonding Failure Location

In the present study most bonding failures were observed in the bracket–composite interface and in the adhesive itself but there was no statistically meaningful relationship between the type of bonding failure in the experimental groups (p=0.532). These findings are consistent with the results of studies carried out by Romano et al. (15). Perdigao et al. (8) reported that bonding failure in the self-etching primer technique is more prevalent in the adhesive and it was attributed to shallow etching in the technique. In the other study, most failures occurred in the dry environment, in bracket–composite interface or inside the adhesive in the conventional and self-etching primer techniques, but in the conventional technique in a saliva-contaminated environment most failures were observed in the enamel–adhesive interface therefore, contamination or lack of contamination with saliva is a major factor in determining bonding failure location. In a study by Romano, using Transbond XT composite, in the conventional bonding and self-etching primer techniques bonding failure was observed in the bracket–adhesive interface, but bonding failure was illustrated in the adhesive–enamel interface with Z-100 and Concise composites. Bracket type, contamination or lack of contamination with saliva and composite type can be mentioned as factors involved in determining bonding failure location.

 Bracket Failure

No cases of bracket failure were observed in the present study. The results of this study are in agreement with those reported by Arici, in which only ceramic brackets have been used (21). An advantage of the present study is the comparison of metallic and ceramic brackets. In a study carried out by Chaconas et al. (21) a number of bracket failures during debonding were observed; the reason behind this was the debonding method in which force was applied to bracket wing because this method increases the risk of bracket failure and is different from the method used in the present study. Some factors involved in bracket failure are bracket type, the method and the equipment/tools used for debonding and the force application location for debonding.

 The Relationship between Bond Strength and ARI

The results found in the present study demonstrated a statistically significant relationship between bond strength changes and ARI changes in all the experimental groups. Bond strength changes in each group were parallel with ARI changes, i.e. bond strength increase or decrease resulted in ARI increase on decrease, respectively.

Under the condition of this investigation:

1. The bond strengths of metallic brackets are considerably higher than ceramic brackets.

2. Self-etching primer technique produces weaker bond strengths, which is not statistically significant but is clinically acceptable in comparison with the conventional technique.

3. Taking ARI into account, there seem to be no concerns regarding enamel damage during debonding with the two bracket types and two bonding types used.

4. Most of the specimens failed at the bracket-adhesive or inside the adhesive itself, which may indicate a reduced chance of enamel damage. A direct and statistically significant relationship was found between bond strength changes and ARI changes.

It is recommended that in the future studies:

1. The results be revaluated in vivo, if possible;

2. Microscopic sections of the debonded area are evaluated under an electron microscope.
